# Detection of selected pathogens in ticks collected from cats and dogs in the Wrocław Agglomeration, South-West Poland

**DOI:** 10.1186/s13071-016-1632-0

**Published:** 2016-06-21

**Authors:** Nina Król, Anna Obiegala, Martin Pfeffer, Elżbieta Lonc, Dorota Kiewra

**Affiliations:** Department of Microbial Ecology and Environmental Protection, Institute of Genetics and Microbiology, University of Wrocław, Przybyszewskiego 63/77, 51-148 Wrocław, Poland; Institute of Animal Hygiene and Veterinary Public Health, University of Leipzig, An den Tierkliniken 1, 04103 Leipzig, Germany

**Keywords:** *Rickettsia* spp., *Anaplasma phagocytophilum*, *Candidatus* Neoehrlichia mikurensis, *Babesia* spp., *Ixodes ricinus*, *Ixodes hexagonus*, *Dermacentor reticulatus*, Ticks, Dogs, Cats

## Abstract

**Background:**

Tick-borne infections are no longer confined to rural areas, they are documented with increasing frequency in urban settlements across the world. They are known to cause diseases in humans as well as in their companion animals.

**Methods:**

During a period of 2 years, from January 2013 until December 2014, ticks were collected from dogs and cats in 18 veterinary clinics in the Wrocław Agglomeration, Poland. In total, 1455 ticks were found on 931 pets: 760 domestic dogs and 171 cats. For molecular examinations 127 *I. ricinus* ticks (115 females and 12 males) were randomly selected, all collected *I. hexagonus* (*n* = 137, 32 females, 98 nymphs, 7 larvae) and all collected *D. reticulatus* (*n* = 46, 31 females, 15 males) were taken. *Ixodes ricinus* and *I. hexagonus* ticks were tested for *Rickettsia* spp., *Anaplasma phagocytophilum, Candidatus* Neoehrlichia mikurensis and *Babesia* spp., while *D. reticulatus* ticks were investigated for *Rickettsia* spp. and *Babesia* spp. only.

**Results:**

In total, 65.4 % *I. ricinus* ticks were infected with at least one pathogen. Over 50 % of *I. ricinus* were positive for *Rickettsia* spp. (*R. helvetica* and *R. monacensis*). The infection level with *A. phagocytophilum* was 21.3 %. DNA of *Cand*. N. mikurensis was detected in 8.1 % *I. ricinus* ticks. Interestingly only female ticks were infected. The prevalence of *Babesia* spp. was confirmed in 9.0 % of *I. ricinus* involving the species *B. microti* and *B. venatorum.* A total of nineteen double, one triple and two quadruple infections were found in *I. ricinus* ticks only. Almost 11 % of *I. hexagonus* ticks were positive for at least one of the tested pathogens. *Rickettsia* spp. infection was found in 2.2 %, while *A. phagocytophilum* was detected in 8.1 % of *I. hexagonus* ticks. Only one nymph was positive for *Cand.* N. mikurensis and none of *I. hexagonus* ticks harbored a *Babesia* spp. Over 60 % of *D. reticulatus* ticks were positive for rickettsial DNA, exclusively belonging to the species *R. raoultii.*

**Conclusion:**

The high tick infestation rates and the prevalence of pathogens found in these ticks demonstrate a serious level of encounter to tick-borne diseases in urban dogs in the Wroclaw area, and provide evidence that dogs and cats themselves may substantially contribute to the circulation of the ticks and pathogens in the urban area.

## Background

Tick-borne pathogens, such as *Anaplasma phagocytophilum*, *Candidatus* Neoehrlichia mikurensis and *Rickettsia* spp., belong to the order Rickettsiales, while such as *Babesia* spp. are parasitic protozoans. All these pathogens, are known to cause diseases in humans as well as in their companion animals, and are considered to be emerging across Europe and other parts of the world [1–6].

*Rickettsia* spp. are divided into four groups: the spotted fever group (SFG), the typhus group, the *Rickettsia bellii* group, and the *Rickettsia canadensis* group [7]. Rickettsiae of the SFG are known to be transmitted by ticks and cause DEBONEL (Dermacentor-borne necrosis erythema lymphadenopathy), also known as TIBOLA (tick-borne lymphadenopathy) in humans [8]. In Poland, rickettsioses caused by rickettsiae of the SFG were described in forest workers, dogs and ticks [9–11].

*Anaplasma phagocytophilum* and *Candidatus* Neoehrlichia mikurensis are gram-negative obligate intracellular bacteria. *Anaplasma phagocytophilum* may cause granulocytic anaplasmosis in humans, dogs, horses and ruminants [4]. Anaplasmosis has been reported in dogs, cats and humans from Poland with evidence of autochthonous human cases [12–15]. Neoehrlichiosis was described in immunodeficient and previously healthy humans but also in immunodeficient dogs [1, 3, 16]. The presence of *Cand*. Neoehrlichia mikurensis has been proven in *Ixodes ricinus* ticks and in asymptomatic humans from Poland [17, 18].

Babesiosis is a zoonotic disease occurring worldwide which is caused by intraerythrocytic parasites of the genus *Babesia* [2]. In Europe, *Babesia divergens*-like organisms are mainly responsible for the disease in humans. *Babesia* spp. are reported in *Ixodes ricinus* and *Dermacentor reticulatus* from Poland [19]. Nowadays, cases caused by tick-borne pathogens are emerging in urban regions in Europe [20, 21]. Dogs and cats should be taken into account as important hosts of ticks in urban areas [21, 22].

In Poland, 5 of the 19 detected tick species (*I. ricinus* Linnaeus, 1758, *I. hexagonus* Leach, 1815, *I. crenulatus* Koch, 1844, *I. rugicollis* Schulze et Schlottke, 1929, and *D. reticulatus* Fabricius, 1794) parasitize on cats and dogs [23]. Most commonly, however, *I. ricinus* and *D. reticulatus*, the important vectors of *Rickettsia* spp., *Anaplasma phagocytophilum* and *Babesia* spp., are detected on pets [21, 24–28]. In a previous study, ticks collected from cats and dogs from the Wrocław Agglomeration, SW Poland, were tested for the presence of *Borrelia* spp. [29].

The aim of this follow-up study was to evaluate the prevalence of *Babesia* spp., *A. phagocytophilum*, *Cand*. Neoehrlichia mikurensis and *Rickettsia* spp. in ticks collected from cats and dogs in the Wrocław Agglomeration, Poland.

## Methods

### Tick collection

During a period of 2 years, from January 2013 till December 2014, ticks were collected from dogs and cats in the veterinary clinics in the Wrocław Agglomeration, Poland. Wrocław city (*c*292.8 km^2^) is located in the south-west of Poland (51°07'N, 17°02'E). Tick collection from 2013 [29] was extended with specimens collected in the next year. In total, 18 veterinary clinics submitted 1455 ticks found on 931 pets: 760 domestic dogs and 171 cats (Table 1). Tick specimens were determined by life stage, sex and species [30]. Tick collection consisted of: 46 *D. reticulatus* ticks (31 females, 15 males), 137 *I. hexagonus* (32 females, 98 nymphs, 7 larvae), and 1272 *I. ricinus* (1160 females, 103 males, 9 nymphs).

**Table 1:** Ticks collected from pets in the Wrocław Agglomeration (Poland), 2013-2014

Species	Number of ticks / number of hosts		
	Cats	Dogs	Total
*Dermacentor reticulatus*	2 / 2	44 / 34	46 / 36
*Ixodes hexagonus*	53 / 7	84 / 37	137 / 44
*Ixodes ricinus*	267 / 162	1005 / 689	1272 / 851
Total	322 / 171	1133 / 760	1455 / 931

### DNA isolation and biological material

All collected ticks were kept in 70 % ethanol until isolation of DNA was performed. Before DNA extraction, ticks were washed in sterile water. All ticks were individually homogenized using sterile polystyrene pistils and then genomic DNA was extracted by using a Tissue Genomic Extraction GPB Mini Kit with proteinase K (Genoplast Biochemicals, Poland) according to the manufacturer’s instructions. All of the obtained lysates were stored at -20 °C until examined.

For further examinations 310 ticks were selected: 127 randomly chosen *I. ricinus* ticks (115 females and 12 males), all collected *I. hexagonus* ticks (*n* = 137; 32 females, 98 nymphs, 7 larvae) and *D. reticulatus* ticks (*n* = 46; 31 females, 15 males) (Table 2). *Ixodes ricinus* and *I. hexagonus* ticks were tested for *Rickettsia* spp., *Anaplasma phagocytophilum, Candidatus* Neoehrlichia mikurensis and *Babesia* spp., while *D. reticulatus* ticks were only investigated for *Rickettsia* spp. and *Babesia* spp.

**Table 2:** Ticks investigated for pathogens, the Wrocław Agglomeration (Poland), 2013–2014

Pathogens	Number of tick stages												
	Females			Males			Nymphs			Larvae			Total
	Collected from												
	Total	Cats	Dogs	Total	Cats	Dogs	Total	Cats	Dogs	Total	Cats	Dogs	
*D. reticulatus*	31	2	29	15	–	15	–	–	–	–	–	–	46
*I. hexagonus*	32	5	27	–	–	–	98	43	55	7	5	2	137
*I. ricinus*	115	34	81	12	4	8	–	–	–	–	–	–	127
Total	178	41	137	27	4	23	98	43	55	7	5	2	310

Molecular detection of *Rickettsia* spp., *Anaplasma phagocytophilum*, *Candidatus* Neoehrlichia mikurensis and *Babesia* spp.

For detection of *Rickettsia* spp., a real-time PCR targeting the *gltA* genome region (70 bp) was used [31]. A real-time PCR targeting *msp2* gene fragment (77 bp) was performed to detect *A. phagocytophilum* [32]. In order to detect *Candidatus* Neoehrlichia mikurensis, a real-time PCR targeting the partial *groEL* gene (99 bp) was used [33, 34]. All PCR methods were carried out using the Mx3000P real-time cycler (Stratagene).

For detection of *Babesia* spp., a conventional PCR amplification of the small 18S subunit of the rRNA gene (411–452 bp) with primers BJ1 and BN2 was performed [35]. Samples positive for *Rickettsia* spp. DNA by real-time PCR were further investigated using a conventional PCR in which a 811-bp fragment of the *ompB* gene was amplified [36]. The PCR products were visualized by electrophoresis on 1.5 % agarose gels stained with Midori Green (NIPPON Genetics, Düren, Germany). Randomly selected positive PCR products (*n* = 22) were purified using the NucleoSpin® and PCR Clean-up Kit (MACHEREY-NAGEL, Düren, Germany) according to the manufacturer’s instructions. Purified PCR products were sequenced (Interdisziplinäres Zentrum für Klinische Forschung, Leipzig, Germany) with forward and reverse primers, and analyzed with Chromas Lite (Technelysium Pty Ltd, Australia). Nucleotide sequences were compared with GenBank entries using NCBI BLAST.

### Statistical analysis

The chi-square test was used to compare infected and not infected ticks (STATISTICA ver. 9.0). Yates’ correction was used for 1-*df* tests when expected frequencies were less than 5. The significance level was set at 0.05.

## Results

The most common infection was *Rickettsia* spp., which was found in 30.6 % (*n* = 95) of all tick species (Table 3). The highest infection level was detected in *D. reticulatus* ticks (60.9 %), followed by *I. ricinus* (50.4 %) and *I. hexagonus* (2.2 %). The differences in prevalence between tick species were statistically significant (*χ*^*2*^ = 95.268, *df* = 2, *P <* 0.001). *Rickettsia raoultii* was found in 100 % sequenced *D. reticulatus* samples (100 % identity to acc. no. JX298077.1). In *I. ricinus* ticks, the distribution of *Rickettsia* spp. was 80 % for *R. helvetica* (100 % identity to acc. no. KR150781.1) and 20 % for *R. monacensis* (100 % identity to KC137254.1). DNA from *I. hexagonus* was not tested due to high CT values previously obtained by RT-PCR (CT > 35). The prevalence of *A. phagocytophilum* was detected in 14.4 % (*n* = 37) of *Ixodes* species. Further, 21.3 % of *I. ricinus*, and 8.1 % of *I. hexagonus* were positive for this pathogen. The infection level was statistically higher in *I. ricinus* ticks than *I. hexagonus* (*χ*^*2*^ = 9.01, *df* = 1, *P =* 0.003). *Candidatus* N. mikurensis was found in 4.2 % of *Ixodes* samples. It was detected in 8.1 % of *I. ricinus* and 0.7 % of *I. hexagonus* (the difference being statistically significant, *χ*^*2*^ = 8.599, *df* = 1, *P =* 0.003). The prevalence of *Babesia* spp. was the lowest among the tested pathogens, 3.6 % (*n* = 11) for all tick species, but only *I. ricinus* ticks (9.0 %) were infected (*χ*^*2*^ = 16.934, *df* = 2, *P <* 0.001). *Babesia microti* was detected in 83.3 % (all samples with identity over 96 % to acc. no. JQ711225.1), and *B. venatorum* in 16.7 % (identical with 99 % to acc. no. KR493907.1 and 98 % to KF500410.1) of sequenced *I. ricinus* samples.

**Table 3:** Ticks collected from dogs and cats infected with pathogens, the Wrocław Agglomeration (Poland), 2013-2014

Pathogens	Number of infected ticks/number of investigated ticks (%)										
	*I. ricinus*			*I. hexagonus*				*D. reticulatus*			TOTAL
	F	M	T	F	N	L	T	F	M	T	
*Rickettsia* spp.	59/115 (51.3)	5/12 (41.7)	64/127 (50.4)	2/32 (6.3)	1/98 (1.0)	0/7 (0.0)	3/137 (2.2)	19/31 (61.3)	9/15 (60.0)	28/46 (60.9)	95/310 (30.6)
*A. phagocytophilum*	25/112 (22.3)	1/10 (10.0)	26/122 (21.3)	2/31 (6.4)	8/97 (8.2)	1/7 (14.3)	11/135 (8.1)	–	–	–	37/257 (14.4)
*Cand.* N. mikurensis	10/113 (8.8)	0/11 (0.0)	10/124 (8.1)	0/32 (0.0)	1/97 (1.0)	0/7 (0.0)	1/136 (0.7)	–	–	–	11/260 (4.2)
*Babesia* spp.	10/112 (8.9)	1/10 (10.0)	11/122 (9.0)	0/31 (0.0)	0/97 (0.0)	0/7 (0.0)	0/135 (0.0)	0/31 (0.0)	0/15 (0.0)	0/46 (0.0)	11/303 (3.6)

*Abbreviations* *: F* females, *M* males, *N* nymphs, *L* larvae, *T* total

*Ixodes ricinus* ticks were more often infected, with minimum one pathogen, than *I. hexagonus* or *D. reticulatus* (*χ*^*2*^ = 90.019, *df* = 2, *P <* 0.001). In total, 65.4 % (*n* = 83) *I. ricinus* ticks were positive for at least a single infection. The most often detected pathogen was *Rickettsia* spp. (*χ*^*2*^ = 84.505, *df* = 3, *P <* 0.0001), in 50.4 % of *I. ricinus* (*n* = 64; Table 3). There were no significant differences in infection levels between females and males (*χ*^*2*^ = 0.404, *df* = 1, *P =* 0.525) nor ticks collected from cats or dogs (*χ*^*2*^ = 0.694, *df* = 1, *P =* 0.405). *Anaplasma phagocytophilum* was verified in 21.3 % specimens (*n* = 26, no statistically significant differences were observed between females and males, *χ*^*2*^ = 0.259, *df* = 1, *P =* 0.611, or between ticks parasitizing cats or dogs, *χ*^*2*^ = 0.002, *df* = 1, *P =* 0.964). *Candidatus* N. mikurensis was detected in 8.1 % *I. ricinus* (*n* = 10), only females were infected (*χ*^*2*^ = 0.202, *df* = 1, *P =* 0.653); infection level of ticks collected from pets was not statistically significant (*χ*^*2*^ = 0.097, *df* = 1, *P =* 0.755). The prevalence of *Babesia* spp. was confirmed in 9.0 % of specimens (n = 11); there were neither significant differences between females and males (*χ*^*2*^ = 0.214, *df* = 1, *P =* 0.644) nor ticks infesting cats or dogs (*χ*^*2*^ = 3,086, *df* = 1, *P =* 0.079).

In total, 10.9 % (*n* = 15) *I. hexagonus* ticks were positive for at least one of the tested pathogens. *Anaplasma phagocytophilum* was the most common infection in these ticks (*χ*^*2*^ = 20.661, *df* = 3, *P <* 0.001), it was detected in 8.1 % of ticks (*n* = 11); no statistically significant differences were observed between life stages (*χ*^*2*^ = 0.473, *df* = 2, *P =* 0.789) or for ticks parasitizing cats or dogs (*χ*^*2*^ = 1.373, *df* = 1, *P =* 0.241). *Rickettsia* spp. infection was found in 2.2 % ticks (*n* = 3), there were no statistically significant differences between life stages, (*χ*^*2*^ = 3.245, *df* = 2, *P =* 0.197) or for ticks from different hosts (*χ*^*2*^ = 0.166, *df* = 1, *P =* 0.684). Only one nymph specimen (0.7 %) was positive for *Cand.* N. mikurensis, with no statistical differences in infection levels between different life stages (*χ*^*2*^ = 0.405, *df* = 2, *P =* 0.817) or ticks collected from pets (*χ*^*2*^ = 0.052, *df* = 1, *P =* 0.82). None of the *I. hexagonus* ticks was found to be infected by *Babesia* spp.

Among *D. reticulatus* only *Rickettsia* spp. was detected; 60.9 % of ticks were positive (*n* = 28). Statistically significant differences were not detected in infection levels between males and females (*χ*^*2*^ = 0.007, *df* = 1, *P =* 0.933) or ticks from cats or dogs (*χ*^*2*^ = 0.175, *df* = 1, *P =* 0.676). *Babesia* spp. DNA was not amplified in any of *D. reticulatus* sample.

Co-infections were detected only in *I. ricinus* ticks, mainly females. Only one male tick had a double-infection with *Rickettsia* spp. and *A. phagocytophilum.* The most common pathogen combination was *Rickettsia* spp. *+ A. phagocytophilum,* followed by  *Rickettsia* spp. *+ Cand.* N. mikurensis, *Rickettsia* spp. *+ Babesia* spp. and one of *Cand.* N. mikurensis + *Babesia* spp. (Table 4). Apart from these, two quadruple-infections and one triple-infection (*Rickettsia* spp., *Cand.* N. mikurensis, *A. phagocytophilum*.)

**Table 4:** Co-infections in *I. ricinus* ticks collected from pets in the Wrocław Agglomeration (Poland), 2013-2014

Co-infections	No. of ticks (collected from cats/dogs)
*Rickettsia* spp. *+ Babesia* spp.	3 (1/2)
*Rickettsia* spp. *+ Cand.* N. mikurensis	4 (1/3)
*Rickettsia* spp. *+ A. phagocytophilum*	11 (3/8)
*Cand.* N. mikurensis + *Babesia* spp.	2 (2/0)
*Rickettsia* spp. *+ Cand.* N. mikurensis + *A. phagocytophilum*	1 (0/1)
*Rickettsia* spp. *+ Cand.* N. mikurensis + *A. phagocytophilum + Babesia* spp.	2 (1/1)

## Discussion

From three ticks species identified as parasites of dogs and cats in the Wrocław Agglomeration, *Ixodes ricinus* was predominant*,* followed by *I. hexagonus* and *D. reticulatus*. Similar findings were obtained in Belgium [37], Switzerland [38], Germany [39] and Great Britain [40], as well as in Bosnia and Herzegovina [41].

The prevalence of pathogens differed between the tick species. *Ixodes ricinus* individuals were the most often infected species. The lowest infection levels were observed in *I. hexagonus* ticks. From all tested pathogens (*Rickettsia* spp., *Babesia* spp., *Candidatus* Neoehrlichia mikurensis and *Anaplasma phagocytophilum*), rickettsial infections were the most common. The infection levels of *A. phagocytophilum, Cand.* N. mikurensis and *Babesia* spp. (*B. microti, B. venatorum*) were the highest in *I. ricinus* ticks and co-infections were detected only in this species. Interestingly, *Babesia* spp. DNA was only found in *I. ricinus* ticks, none of the *D. reticulatus*, the main vector for *B. canis* [42], was infected. The most often detected pathogen in *I. hexagonus* specimens was *A. phagocytophilum.* There were no statistically significant differences in infection levels between different tick life stages and between ticks collected from cats and dogs in any case. In Switzerland [43], researchers observed a difference in *Rickettsia* spp. infection levels between ticks from cats (40 %) and dogs (18 %), which was not found in this study.

*Rickettsia* spp. was dominant among *D. reticulatus* ticks (only *R. raoultii*) with prevalence over 60 % and *I. ricinus* (only *R. helvetica* and *R. monacensis*) - over 50 %. Only 2 % of *Ixodes hexagonus* ticks were infected. In Europe, prevalence of *Rickettsia* spp. in ticks infesting urban animals reached levels, e.g. 14–61 % in *I. ricinus,* 1–44 % of *I. hexagonus*, 14–39 % of *D. reticulatus* [37, 44–46]. In Poland, the infection levels in the questing *I. ricinus* ticks were much lower than results obtained in this study; they differed from 6 to 23 % [47, 48] but prevalence in *D. reticulatus* was similar, 57 % [49].

*Anaplasma phagocytophilum* infection levels of *I. ricinus* in this research were comparable to those reported in Belgium but much lower for *I. hexagonus* [37]. However, in The Netherlands and Germany, the prevalence was lower in *I. ricinus* ticks but similar to *I. hexagonus* [44, 50]. In Poland, also in Lower Silesia, the infection of *I. ricinus* ticks (questing as well as collected from dogs) was lower than the data presented here [51–53].

*Candidatus* N. mikurensis was previously found in ticks in Poland [18], however, the detection in feeding *I. hexagonus* is the first one in the country. In Germany, the prevalence of this pathogen in *I. ricinus* and *I. hexagonus* from pets was 4 % and 6 %, respectively [45], while in Denmark only 1 % of *I. ricinus* was infected [46]. The prevalence in *I. hexagonus* ticks in the present study (0.7 %) is lower than in other countries; however, it is comparable to results obtained in a former study from Poland conducted on other tick species, *I. ricinus* (0.5 %) [18].

The prevalence of *Babesia* spp. (*B. microti* and *B. venatorum*) in *I. ricinus* was higher than in Belgium [54] and Germany [45], where the infection was also detected in *I. hexagonus* ticks. In Poland, also in Lower Silesia, only 1–3 % of questing *I. ricinus* ticks were infected [52, 53, 55]. Similar to our results, all tested *D. reticulatus* in Germany were free of babesial parasites [45]*.* However, 11 % of *D. reticulatus* ticks infesting dogs in central Poland were infected [51]; in Austria 2 of 6 specimens and in Hungary almost 30 % of *D. reticulatus* (only female ticks) were positive for *B. canis* [56, 57].

As results of this study show, the risk of tick-borne diseases (TBD) is high in the Wrocław Agglomeration. However, due to the limitation of this study (no blood samples of dogs and cats were investigated for the pathogens), the tick-borne situation among pets in this area is not fully estimated. In Poland, canine tick-borne diseases pose an emerging veterinary problem. The most common TBD among dogs are *B. canis* and *A. phagocytophilum* reaching levels of 28 and 12 %, respectively [58, 59]. Apart from the above, dogs were infected with *Borrelia burgdorferi* (*s.l*.), and *Ehrlichia canis.*

## Conclusion

The high infection levels were detected for *Rickettsia* spp. (*R. raoultii, R. helvetica* and *R. monacensis), Anaplasma phagocytophilum, Candidatus* Neoehrlichia mikurensis and *Babesia* spp. (*B. microti* and *B. venatorum)* in ticks infesting dogs and cats in the Wrocław Agglomeration, Poland. These findings, as well as the high tick infestation rates, demonstrate a serious level of encounter to tick-borne diseases in urban dogs and cats in the Wrocław area, and provide evidence that dogs and cats themselves may substantially contribute to the circulation of the ticks and the pathogens in the urban area.

## Abbreviations

DEBONEL, dermacentor-borne necrosis erythema lymphadenopathy; SFG, spotted fever group; TBD, tick-borne disease; TIBOLA, tick-borne lymphadenopathy
